# Outcomes From Cytotoxic Chemotherapy Following Progression on Immunotherapy in Metastatic Melanoma: An Institutional Case-Series

**DOI:** 10.3389/fonc.2022.855782

**Published:** 2022-04-28

**Authors:** Elizabeth M. Gaughan, Bethany J. Horton

**Affiliations:** ^1^ Department of Medicine, Division of Hematology and Medical Oncology, University of Virginia, Charlottesville, VA, United States; ^2^ Department of Public Health Sciences, University of Virginia, Charlottesville, VA, United States

**Keywords:** chemotherapy, immunotherapy, progression, metastatic melanoma, cancer

## Abstract

**Introduction:**

The role of chemotherapy in the management of advanced melanoma is limited due to low response rates and short survival. Improved outcomes to chemotherapy administered after immunotherapy for metastatic melanoma and other solid tumors have been reported. We studied the outcomes of subjects treated at the University of Virginia (UVA) with chemotherapy following progression on prior systemic immunotherapy and compared the results with the existing literature.

**Materials and Methods:**

Subjects were identified through an institutional database of patients treated with immunotherapy at UVA. Demographic, pathologic and clinical factors were collected, along with dates of therapy, investigator-assessed best response as per Response Evaluation Criteria for Solid Tumors version 1.1 and dates of death or last follow up. Kaplan-Meier survival estimates and log-rank tests were used to perform time to event analysis of progression free survival and overall survival.

**Results:**

Forty-five patients were identified who met the inclusion criteria including 24 men and 21 women with a median age of 61 years. All patients had received at least one line of immunotherapy including 64.4% with prior anti-PD1 treatment. The cytotoxic chemotherapy regimens used included carboplatin with paclitaxel (55.6%), temozolomide (31.1%) and *nab-*paclitaxel (13.3%). The overall response rate for cytotoxic chemotherapy 22.2% and the disease control rate was 35.6%. The median progression-free survival was 1.7 months and median overall survival was 4.7 months. Nineteen (42.2%) patients survived greater than 6 months and seven (15.5%) patients survived over 12 months. Fourteen patients were able to proceed to further therapy.

**Discussion:**

Our results reveal that receipt of immunotherapy prior to chemotherapy for metastatic melanoma does not appear to improve the benefit of chemotherapy. The palliation of symptoms, maintenance of performance status and disease control may be valuable for some patients during this time of robust research and discovery for metastatic melanoma.

## Introduction

Advances in immunotherapy and targeted therapy have revolutionized the management of metastatic melanoma. Despite the prolonged responses and improvements in survival seen with these treatments, many patients ultimately progress and seek additional therapy. The role of chemotherapy for melanoma remains limited and uncertain, with the agents often used in the late disease setting after failure of or ineligibility for other treatment. The relatively rapid pace of research and development of new and effective therapies for melanoma raises the value of disease control and the maintenance of performance status through palliation of cancer-related symptoms. Treatments that can offer these outcomes, such as chemotherapy, may help some patients in the salvage setting access emerging therapies.

Most data on chemotherapy for melanoma come from studies conducted before the widespread use of immune checkpoint inhibition and BRAF-targeted agents. There is a long history of utilizing the alkylating agents dacarbazine and temozolomide in this setting, with overall response rates (ORR) of 7.2-21%, median progression free survival (mPFS) of 1.5-2.3 months and overall survival (OS) of 5.5-10.8 months (1–5). More recently, *nab*-paclitaxel demonstrated single-agent activity in advanced melanoma patients with an ORR of 15-21.6% and mOS of 9.6-12.6 months (6, 7). The most common combination regimen is carboplatin and paclitaxel with an ORR of 11-20%, and mOS 8.6-11.3 months (8–10). Overall, these data indicate that chemotherapy can provide response in some patients with a limited impact on survival.

The effect of prior immunotherapy on the chemotherapy outcomes of patients with advanced melanoma has not been prospectively studied. Retrospective case series suggest the potential for improved responses and survival from chemotherapy treatment after immunotherapy for melanoma and other solid tumors (11–20). Our institutional experience also revealed some patients with unexpected and notable benefit to chemotherapy following progression on immunotherapy including patients who were able to access new melanoma therapy after disease stabilization. We studied the outcomes of patients treated at the University of Virginia (UVA) with chemotherapy after progression on prior systemic immunotherapy and compared the results with the existing literature.

## Materials and Methods

After obtaining UVA -Institutional Review Board (IRB) approval, subjects were identified through an IRB-approved institutional database of patients treated with immunotherapy. Patients were included if they received immunotherapy in the advanced disease setting (metastatic or unresectable melanoma), including interleukin-2, ipilimumab, ipilimumab and nivolumab combination, pembrolizumab and nivolumab, followed by the receipt of cytotoxic chemotherapy. Included patients may have received any number of regimens of immunotherapy and/or targeted therapy prior to chemotherapy administration. Any regimen of chemotherapy administered in the second line or beyond for advanced melanoma was allowed, including single-agent and combination treatments. For subjects that received multiple lines of chemotherapy, only data for the first line of chemotherapy were collected. For each subject, data on demographics, melanoma characteristics, staging per AJCC 7^th^ edition and prior treatment history were obtained. The type of chemotherapy, treatment course, investigator-assessed best response to therapy *via* Response Evaluation Criteria in Solid Tumors version 1.1 criteria and date of progression were collected.

Kaplan-Meier survival estimates and log-rank tests were used to perform time to event analyses of progression free survival and overall survival. Standard descriptive statistics were used to summarize baseline patient characteristics. ORR is defined as the percentage of subjects experiencing a complete response (CR) or partial response (PR) as their best response at any time, reported by the investigator. Disease control rate (DCR) is defined as the percentage of patients with CR, PR or stable disease (SD) as their best response at any time, reported by the investigator. Progression-free survival (PFS) is calculated as time from the start of chemotherapy to progression. Overall survival (OS) is calculated as the time from the start of chemotherapy to either death or last follow-up date, if a date of death is unavailable. All analyses were performed using SAS 9.4 (Cary, NC).

## Results

Of the 549 patients with advanced melanoma treated at UVA with immunotherapy from 01/01/2011 through 04/05/2021, 45 met inclusion criteria. Of these, 53.3% were male, 95.6% were white and the median age at advanced melanoma diagnosis was 61 years (**Table 1**). Most patients, 31 (68.9%) had a cutaneous primary, while seven (15.6%) had a mucosal, six (13.3%) had uveal and one (2.2%) had conjunctival primary melanoma. Most, 31 (68.9%) had wild-type tumors, while seven patients had tumors with *BRAF* V600E mutation (15.6%). Twelve subjects (26.7%) had a history of brain metastases, 39 (86.7%) had M1c disease and 23 (51.1%) had an elevated lactate dehydrogenase.

**Table 1:** Patient characteristics.

DEMOGRAPHICS	N = 45 patients (percentage)
Gender	
Male	24 (53.3%)
Female	21 (46.7%)
Race	
White	43 (95.6%)
African American	1 (2.2%)
Other	1 (2.2%)
Median Age at Diagnosis	61 years (range 21-86 years)
**DISEASE CHARACTERISTICS**	
Type of Primary Melanoma	
Cutaneous	31 (68.9%)
Mucosal	7 (15.6%)
Ocular	6 (13.3%)
Conjunctival	1 (2.2%)
Mutation	
* BRAF* V600E	7 (15.6%)
* NRAS*	2 (4.4%)
c-KIT	1 (2.2%)
Wild-Type	31 (68.9%)
Not Reported	4 (8.9%)
Brain Metastases	
Yes	12 (26.7%)
No	33 (73.3%)
LDH ≥ than upper limit of normal	
Yes	23 (51.1%)
No	20 (44.4%)
Not Reported	2 (4.4%)
Stage (AJCC 7^th^ Edition)	
Unresectable III	3 (6.7%)
M1a	1 (2.2%)
M1b	2 (4.4%)
M1c	39 (86.7%)
**TREATMENT HISTORY**	
Lines of Prior Therapy (1-7)	
1	9 (20.0%)
2	19 (42.2%)
3	10 (22.2%)
4	5 (11.1%)
5	1 (2.2%)
7	1 (2.2%)
Lines of Prior Immunotherapy (1-4)	
1	10 (22.2%)
2	21 (46.7%)
3	11 (24.4%)
4	3 (6.7%)
Types of Prior Immunotherapy	
Interleukin-2	20 (44.4%)
Ipilimumab monotherapy	21 (46.7%)
Combination	16 (35.6%)
Ipilimumab/Nivolumab	
Anti-PD1 monotherapy	29 (64.4%)
Other	5 (11.1%)
Prior Targeted Therapy	8 (17.8%)

All patients had received at least one line of immunotherapy prior to chemotherapy, including interleukin-2, ipilimumab monotherapy, anti-PD1 monotherapy, and ipilimumab and nivolumab combination (**Table 1**). The median time from diagnosis of metastatic disease to initiation of chemotherapy was 14.1 months. Patients received up to seven lines of prior treatment, including up to four lines of prior immunotherapy before chemotherapy administration. Twenty-nine (64.4%) subjects received at least one line of anti-PD1 monotherapy with pembrolizumab or nivolumab and 16 (35.6%) subjects received combination ipilimumab and nivolumab. There were 16 subjects in the cohort without prior exposure to anti-PD1 therapy including three patients without any prior immune checkpoint inhibitor treatment. Twenty patients (44.4%) had received prior interleukin-2.

**Figure 1:**
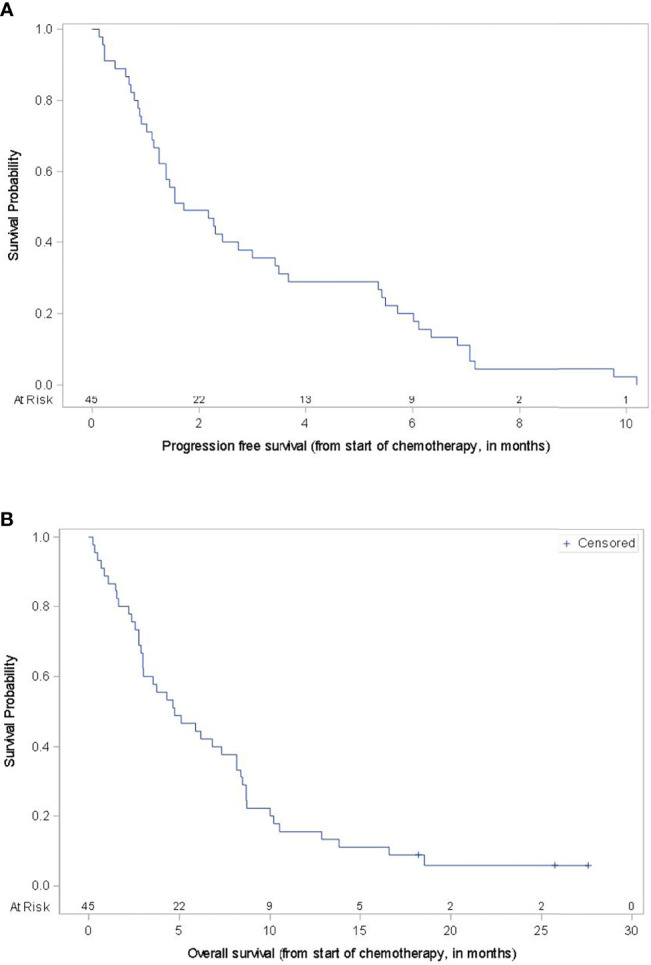
Progression-Free Survival and Overall Survival. Kaplan-Meier survival curves for progression-free survival **(A)** and overall survival **(B)** for the entire cohort.

Patients received one of the following chemotherapy regimens: carboplatin with paclitaxel (55.6%), *nab*-paclitaxel (13.3%) or temozolomide (31.1%) (**Table 2**). No subject received concurrent chemotherapy and immunotherapy. For the overall cohort, ten subjects achieved a partial response to therapy (22.2%), while six subjects (13.3%) had stable disease, leading to an overall disease control rate (DCR) of 35.6%. The ORR to chemotherapy ranged 14.3% to 28%, and the DCR ranged 28.6% to 40% depending on the regimen administered with the highest response rates seen with carboplatin and paclitaxel. In this dataset, no patient experienced a complete response and all patients ultimately experienced disease progression. Three patients were censored at their last follow up date due to unavailable date of death (1 patient) and the patient being alive at the time of analysis (2 patients). The mPFS for the cohort was 1.7 months and mOS was 4.7 months (**Figure 1**). There was no statistically significant difference in mPFS or mOS observed across the different chemotherapy types (log-rank p-values 0.8366 and 0.1889, respectively). Nineteen (42.2%) patients survived greater than 6 months after starting chemotherapy and seven (15.5%) patients survived over 12 months. Fourteen subjects (31.1%) went onto subsequent lines of therapy. All of these 14 patients had received prior Ipilimumab either monotherapy or in combination with nivolumab and 12 had received both prior Ipilimumab and anti-PD1 therapy. Eleven of the 14 did not have brain metastases, 11 had *BRAF* wild-type tumors and 10 were treated with carboplatin and paclitaxel.

**Table 2:** Chemotherapy outcomes.

	N	%	ORR (%)	DCR (%)	mPFS (months) (95% CI)	mOS (months) (95% CI)
**OVERALL COHORT**	45	100	22.2	35.6	1.7 (1.2, 3.0)	4.7 (3.0, 8.2)
**CHEMOTHERAPY REGIMEN**						
Carboplatin/Paclitaxel	25	55.6	28	40	1.5 (1.2,5.4)	5.9 (2.9,8.7)
*nab*-Paclitaxel	6	13.3	16.7	33.3	2.4 (0.9,6.1)	6.0 (2.8,–)
Temozolomide	14	31.1	14.3	28.6	2.0 (0.7,3.4)	3.4 (1.1,8.2)
**BRAF MUTATION STATUS**						
BRAF V600E positive	7	15.6	14.3	28.6	1.2 (0.2,5.4)	2.9 (0.3,6.8)
BRAF wild-type/not-reported	38	84.4	23.7	36.8	2.2 (1.2,3.4)	5.5 (3.0,8.4)
**BRAIN METASTASIS**						
Positive	12	26.7	33.3	50	2.9 (0.2,6.8)	4.4 (0.7,10.0)
Negative	33	73.3	18.2	30.3	1.5 (1.2,2.4)	4.7 (2.9,8.4)
**TYPE OF PRIMARY LESION**						
Cutaneous	31	68.9	25.8	35.5	1.7 (1.2,3.7)	4.3 (2.8,8.2)
Mucosal	7	15.6	14.3	28.6	0.9 (0.4,5.4)	4.6 (1.1,8.5)
Ocular	6	13.3	0.0	33.3	2.8 (1.0,6.0)	7.3 (3.0,–)
Conjunctival	1	2.2	100	100	3.0 (–)	5.1 (–)
**PRIOR ANTI-PD1**						
Positive	29	64.4	20.7	37.9	1.5 (1.2,3.5)	5.9 (3.0,8.7)
Negative	16	35.6	25.0	31.3	2.3 (0.7,3.7)	3.4 (1.1,8.2)

Response rate and survival results for the entire cohort and based on clinical and pathologic variables of interest.

**Figure 2:**
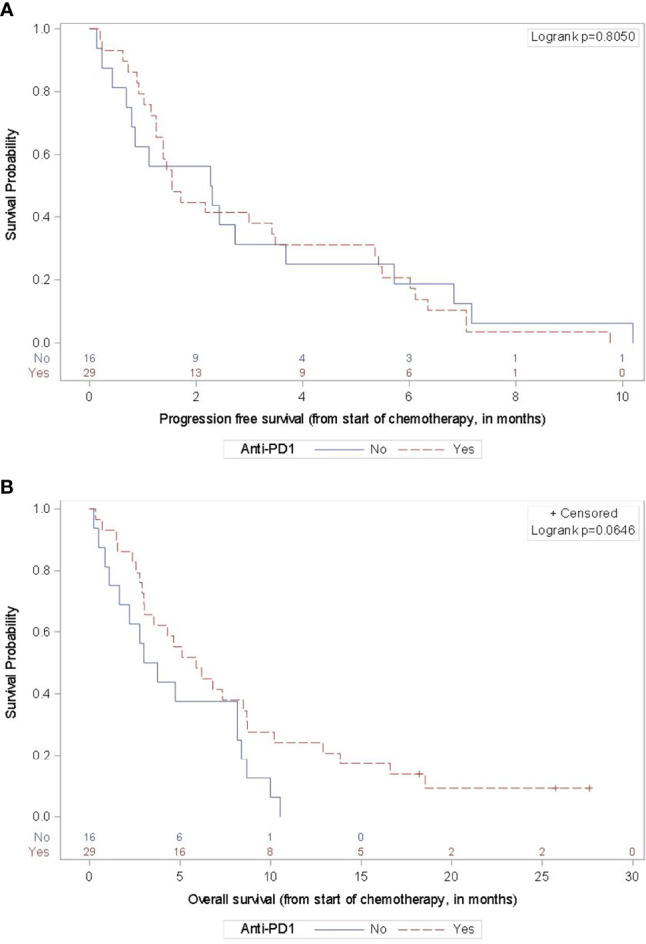
Progression-Free and Overall Survival by Prior Anti-PD-1 Therapy. For the 29 subjects with any prior anti-PD1 therapy in the advanced disease setting, the mPFS **(A)** was 1.5 months (95% CI 1.2-3.5 months) and the mOS **(B)** was 5.9 months (95% CI 3.0-8.7). For the 16 subjects without prior anti-PD1 therapy, the mPFS was 2.3 months (95% CI 0.7-3.7 months) and the mOS was 3.4 months (95% CI 1.1-8.2 months).

The response and survival outcomes based on clinical and pathologic features are outlined in **Table 2**. A history of anti-PD1 therapy prior to chemotherapy resulted in a marginally better overall survival versus those without prior anti-PD1 exposure (mOS 5.9 versus 3.4 months, p=0.0646) (**Figure 2**). Patients with *BRAF-*mutant tumors had numerically worse survival than those with *BRAF-*wild-type tumors or tumors of unknown *BRAF* mutation status (mOS 2.9 versus 5.5 months, p=0.4565), though not statistically significant. The ORR for subjects with primary cutaneous melanoma was numerically highest of the primary sites and within the cutaneous melanoma subgroup, there was slightly higher ORR and DCR for patients with prior anti-PD1 exposure versus no prior anti-PD1 treatment (27.8% vs 23.1% and 38.9% vs 30.8%, respectively) without a difference in survival.

## Discussion

Chemotherapy has long played a limited role in the management of melanoma, typically utilized in the resistant/refractory setting or for patients with contraindication to immunotherapy. Modern treatment options including immune checkpoint inhibitors and targeted therapy result in improved outcomes for most patients. Despite the durable responses experienced by some patients, many will progress and there is a continued need for additional therapy. The success of checkpoint inhibitors and targeted therapy, coupled with the relatively low toxicity burden, often results in patients with preserved performance status that permits further therapy. While clinical trial participation to test new therapies and combinations is a priority, access to these treatments is limited. Cytotoxic chemotherapy is often readily available and understanding optimal value of the agents can be useful for counseling patients and maximizing benefit.

Our own institutional experience, and others, reveals that some melanoma patients have an exceptional response to chemotherapy after progression on immunotherapy (11–15). Our observed clinical scenarios involve responses leading to significant palliation, disease control lasting sufficiently until a new agent became available and responses >1 year in some patients. Maeda et al, presented their retrospective analysis of seven melanoma patients that received at least two cycles of carboplatin and paclitaxel after progression on immune checkpoint inhibition in Japan. They showed a 29% ORR, 57% DCR, mean PFS of 5 months and mean OS of 7.6 months (12). In 2020, Hadash-Bengad et al, published their single-center retrospective assessment of patients treated with chemotherapy (dacarbazine, temozolomide or carboplatin with paclitaxel) after immunotherapy (n=11) versus those who received chemotherapy without prior immunotherapy (n=24) in Israel (11). The mPFS for the post-immunotherapy cohort was 5.2 months versus the 2.5 months in the no-prior immunotherapy cohort (p=0.039). The mOS result (11.8 months versus 8.6 months) and the response rate difference (36.4% versus 19%) were not statistically significant. Also in 2020, Saint-Jean et al. reported their institutional experience of 18 subjects who received chemotherapy (dacarbazine alone or in combination with carboplatin or fotemustine) after failure or limiting toxicity of prior immunotherapy in France (13). They showed a 19% ORR and 25% DCR, with a mPFS of 5.4 months and mOS of 12 months. Taken together, these reports are suggestive of higher response rate and slightly longer survival with chemotherapy than the prospective studies. The cohorts were small, and patients received a variety of chemotherapeutic agents, limiting interpretation of results.

While there were individual patients with notable benefit, the results for our cohort are similar to the historic experience with chemotherapy. Our ORRs ranging 14.3%-28% depending on the regimen used, are in line with prospective trials results with temozolomide and *nab*-paclitaxel and slightly higher than ORRs reported for combination carboplatin and paclitaxel (4–10). Our overall mPFS of 1.7 months and mOS of 4.7 months are numerically lower in comparison with historical controls (1–10). This cohort of patients included subjects with cutaneous, mucosal or uveal melanoma and any number of prior treatments in the advanced disease setting was permitted. Patients were identified through a clinical database of all patients treated with immunotherapy at UVA since 2011, and therefore, representative of the real-world, varied patient population seen over 10 years. Many of the comparison prospective studies excluded patients with uveal melanoma and limited the number of prior systemic agents. Only three subjects in our entire cohort had only received interleukin-2 and had no exposure to immune checkpoint inhibition prior to chemotherapy. Sixteen patients did not receive prior anti-PD1 therapy (either monotherapy or in combination with ipilimumab) before receipt of chemotherapy. While the response rate and mPFS were similar for those with and without prior anti-PD1 exposure, there was a marginally better mOS for patients with prior anti-PD1 treatment.

Forty-two percent of our cohort survived greater than 6 months after chemotherapy and 16% survived greater than 12 months. Fourteen patients were able to go onto subsequent treatment after progression on chemotherapy. Two subjects were alive past the data cutoff, 18 and 28 months after chemotherapy administration. Both subjects experienced partial response to chemotherapy and were able to access additional effective agents after progression. It is difficult to know if these subjects had greater benefit to chemotherapy because of their prior immunotherapy or if their tumors would have been sensitive to the chemotherapy regardless of prior treatment.

The strengths of our analysis include the size and full scope of our single institution experience over the last 10 important years of melanoma therapy advancement. It provides a real-world population for analysis with various types of primary melanoma, presence of brain metastases and high-stage disease, and a variety of prior immunotherapy agents including cytokines, checkpoint inhibitors and investigational vaccine therapy. All clinical, pathologic and radiographic data was available to the investigators for review which standardized interpretation. The limitations of our data include the retrospective nature of the analysis and the lack of biologic correlates for the outcomes. There was no standard time to chemotherapy administration, with a range of 2.0 to 99.2 months after the diagnosis of metastatic disease. Subjects had up to seven lines of prior systemic therapy for advanced disease reflecting the biologic diversity of the tumors under evaluation.

Our results reveal that receipt of immunotherapy prior to chemotherapy for metastatic melanoma does not appear to improve the benefit of chemotherapy. The opportunity to palliate symptoms, maintain performance status and disease control can be valuable during this time of research and discovery for metastatic melanoma.

## Data Availability Statement

The raw data supporting the conclusions of this article will be made available by the authors, without undue reservation.

## Ethics Statement

The studies involving human participants were reviewed and approved by University of Virginia Institutional Review Board. Written informed consent for participation was not required for this study in accordance with the national legislation and the institutional requirements.

## Author Contributions

EG: study concept and design, subject identification, data collection, data interpretation and manuscript preparation. BH: study design and statistical data interpretation. All authors contributed to the article and approved the submitted version.

## Funding

This work was supported by the UVA Cancer Center Support Grant P30CA044579.

## Conflict of Interest

The authors declare that the research was conducted in the absence of any commercial or financial relationships that could be construed as a potential conflict of interest.

The handling Editor declared a past co-authorship with one of the authors EG.

## Publisher’s Note

All claims expressed in this article are solely those of the authors and do not necessarily represent those of their affiliated organizations, or those of the publisher, the editors and the reviewers. Any product that may be evaluated in this article, or claim that may be made by its manufacturer, is not guaranteed or endorsed by the publisher.
